# Resistance to Spot Blotch in Two Mapping Populations of Common Wheat Is Controlled by Multiple QTL of Minor Effects

**DOI:** 10.3390/ijms19124054

**Published:** 2018-12-14

**Authors:** Pawan Kumar Singh, Xinyao He, Carolina Paola Sansaloni, Philomin Juliana, Susanne Dreisigacker, Etienne Duveiller, Uttam Kumar, Arun Kumar Joshi, Ravi Prakash Singh

**Affiliations:** International Maize and Wheat Improvement Center (CIMMYT), Apdo. Postal 6-641, Mexico DF 06600, Mexico; x.he@cgiar.org (X.H.); C.Sansaloni@cgiar.org (C.P.S.); P.JULIANA@cgiar.org (P.J.); S.Dreisigacker@CGIAR.ORG (S.D.); E.Duveiller@cgiar.org (E.D.); U.Kumar@cgiar.org (U.K.); a.k.joshi@cgiar.org (A.K.J.); R.SINGH@CGIAR.ORG (R.P.S.)

**Keywords:** *Triticum aestivum*, *Cochliobolus sativus*, *Bipolaris sorokiniana*, QTL mapping, resistance breeding

## Abstract

Spot blotch (SB) is an important fungal disease of wheat in South Asia and South America. Host resistance is regarded as an economical and environmentally friendly approach of controlling SB, and the inheritance of resistance is mostly quantitative. In order to gain a better understanding on the SB resistance mechanism in CIMMYT germplasm, two bi-parental mapping populations were generated, both comprising 232 F_2:7_ progenies. Elite CIMMYT breeding lines, BARTAI and WUYA, were used as resistant parents, whereas CIANO T79 was used as susceptible parent in both populations. The two populations were evaluated for field SB resistance at CIMMYT’s Agua Fria station for three consecutive years, from the 2012–2013 to 2014–2015 cropping seasons. Phenological traits like plant height (PH) and days to heading (DH) were also determined. Genotyping was performed using the DArTSeq genotyping-by-sequencing (GBS) platform, and a few D-genome specific SNPs and those for phenological traits were integrated for analysis. The most prominent quantitative trait locus (QTL) in both populations was found on chromosome 5AL at the *Vrn-A1* locus, explaining phenotypic variations of 7–27%. Minor QTL were found on chromosomes 1B, 3A, 3B, 4B, 4D, 5B and 6D in BARTAI and on chromosomes 1B, 2A, 2D and 4B in WUYA, whereas minor QTL contributed by CIANO T79 were identified on chromosome 1B, 1D, 3A, 4B and 7A. In summary, resistance to SB in the two mapping populations was controlled by multiple minor QTL, with strong influence from *Vrn-A1*.

## 1. Introduction

Spot blotch (SB) is an important fungal disease in wheat with global importance, mostly prevailing in humid tropical regions of South Asia and South America (such as eastern India, Bangladesh, Nepal, Brazil, Paraguay and Bolivia) and Zambia [1]. Yield losses due to this disease vary from 11 to 52% under various environmental conditions [2]. The causal agent *Cochliobolus sativus* (Ito and Kurib.) Drechsler ex Dastur (anamorph *Bipolaris sorokiniana* (Sacc.) Shoem.) is a semi-biotrophic pathogen with a worldwide distribution [3]. It is the anamorph that causes severe foliar blotch in field conditions, whereas the teleomorph (the sexual stage) has only been found under natural environments in Zambia [4]. 

A range of management strategies have been proposed and applied to prevent the disease, including timely sowing, adequate fertilization, chemical control, crop rotation and tillage system [5]; but host resistance has always been regarded as an indispensable component in the disease management system. Resistance to SB is of quantitative nature and is subjected to genotype-by-environment interaction [6,7]. Early marker-trait association studies showed several markers to be linked with SB resistance, like simple satellite repeat (SSR) markers *gwm67*, *gwm570* and *gwm469* [8]; however recent QTL mapping studies provided more detailed information on the chromosome positions of SB resistance QTL and their effects. In the Chinese resistant cultivar “Yangmai#6”, Kumar et al. [9] identified four QTL on chromosomes 2AL, 2BS, 5BL and 6DL. Soon afterwards, the same authors reported SB resistance QTL on chromosomes 2AS, 2BS, 5BL and 7DS in the cultivar “Ning 8201” and on chromosomes 2BS, 2DS, 3BS, 7BS and 7DS in the cultivar “Chirya#3” [10]. In a mapping population derived from the cross Avocet × Saar, Lillemo et al. [11] demonstrated the major effect of pleiotropic multi-fungal resistance gene *Lr34* located on chromosome 7DL and the minor effect of gene *Lr46* located on chromosome 1BL on SB resistance, the former gene being named as *Sb1*. This study additionally unraveled the underlying mechanism for the phenotypic association between SB resistance and leaf tip necrosis (LTN) earlier reported by Joshi et al. [12], since both *Lr34* and *Lr46* are associated with LTN. In a CIMMYT synthetic wheat derived line SYN1, Zhu et al. [13] identified QTL for SB resistance on chromosomes 1B, 3BS and 5AL. The major QTL on chromosome 5BL was reported by Kumar et al. [9,10] and was recently designated as *Sb2* [14]. Another major QTL on chromosome 3BS was fine mapped and designated as *Sb3* [15]. Additionally, Singh et al. [16] reported two major QTL on chromosomes 7BL and 7DL in a Brazilian SB resistant cultivar “BH 1146”, and Adhikari et al. [17] and Gurung et al. [18] identified multiple QTL on various chromosomes using association mapping. 

Recently, a virulence gene *ToxA* that exists in both *Pyrenophora tritici-repentis* (the pathogen of tan spot, TS) and *Parastagonospora nodorum* (the pathogen of Septoria nodorum blotch, SNB) was found in several Australian isolates of *B. sorokiniana* [19]. The gene is well known to interact with a wheat gene *Tsn1*, conferring susceptible reaction to TS and SNB; hence, it might play significant roles in the spot blotch-*B. sorokiniana* pathosystem as well. Friesen et al. [20] demonstrated the presence of *ToxA* in some *B. sorokiniana* isolates collected from southcentral Texas, USA, and inoculation experiments at seedling stage demonstrated major effects of the *Tsn1* locus on 5BL. Nevertheless, the importance of *Tsn1* in SB resistance under field condition remains unknown. 

Association of several morphological and phenological traits with SB resistance including erect leaf posture, LTN, stay green, days to heading (DH) and plant height (PH) has also been studied [5,21,22]. DH and PH often show negative correlation with SB severity, or late and tall genotypes tend to have low SB infection, although early and short lines resistant to SB have also been reported [22,23]. Similar associations have been reported for Fusarium head blight [24,25] and Septoria tritici blotch [26], which is generally ascribed to disease escape, although other underlying mechanisms may have also contributed to the associations. 

Breeding for SB resistance is one of the major targets of wheat improvement at the International Maize and Wheat Improvement Center (CIMMYT). Large-scale germplasm screening for SB resistance was performed at CIMMYT in 1980s and 1990s, and the resultant resistant lines were used extensively in CIMMYT’s wheat breeding programs [27]. Resistant materials from relatives of common wheat and/or their derivatives were made available [28]. In order to facilitate the utilization of SB resistant materials by breeders and researchers worldwide, a special nursery called CSISA-SB was developed in 2009, which included elite CIMMYT breeding lines with promising SB resistance, as well as good agronomy and high yield potential [23]. The nursery has been sent to multiple South Asian and South American countries where SB is of major importance, as well as to other countries where the disease is found. In 2015, the nursery was renamed as Helminthosporium Leaf Blight Screening Nursery (HLBSN) but the numbering was in accordance to the previous CSISA-SB nurseries, thus the 7th HLBSN was the first HLBSN released.

In order to gain a better understanding of the SB resistance mechanism in CIMMYT germplasm, two CIMMYT resistant lines, BARTAI and WUYA, were crossed with the susceptible line CIANO T79 to develop mapping populations for the dissection of SB resistance factors.

## 2. Results

### 2.1. Phenotypic Analysis

**Figure 1:**
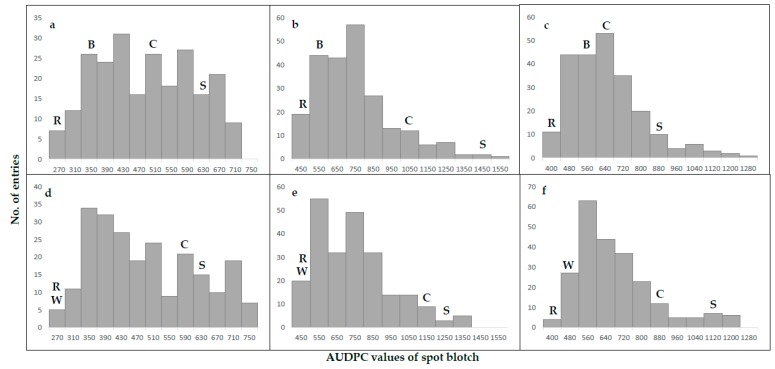
Frequency distribution of AUDPC values of spot blotch for the “BARTAI” × “CIANO T79” (**a**–**c**) and “WUYA” × “CIANO T79” (**d**–**f**) populations in 2013 (**a**,**d**), 2014 (**b**,**e**) and 2015 (**c**,**f**). ***B*** BARTAI, ***W*** WUYA, ***C*** CIANO T79, *R* the resistant check CHIRYA#3, and *S* the susceptible check SONALIKA.

Disease pressure varied significantly among cropping seasons in both populations, with the “year” factor contributing the largest portion to the phenotypic variation. The “genotype” and “genotype × year” factors were also highly significant (Table 1). Both populations reacted similarly within single years, and showed the highest infection rate in 2014 and the lowest rate in 2013; nevertheless, the lines were segregating well with a continuous distribution found in all experiments (Figure 1). The resistant parents and CIANO T79 were always on the resistant and susceptible side of the distribution, respectively; but transgressive segregation consistently occurred in both directions (Figure 1). Specifically, 38 (16.4%) lines in the BARTAI × CIANO T79 (referred as the BC population hereafter) showed an averaged AUDPC value smaller than 446, the score of the resistant parent BARTAI; one of them had a score of merely 278, even better than the resistant check “Chirya#3” with a score of 297. In the WUYA × CIANO T79 (the WC population), however, only 7 (3.0%) lines performed better than the resistant parent and none was better than “Chirya#3”. High broad-sense heritability estimates of SB resistance from 0.86 to 0.88 were found for the two populations (Table 1). Significant correlations were found for AUDPC values among years, with Pearson correlation coefficients ranging from 0.67 to 0.77 in the BC popualtion and from 0.70 to 0.78 in the WC population.

DH and PH always showed negative correlations with SB severity. The late maturing and taller lines tended to have lower SB infection; but the correlations between DH and SB were mostly significant and showed higher correlation coefficients compared to those between PH and SB (Table 2).

### 2.2. Genotyping and Linkage Analysis

Initially about 18,000 GBS markers were scored for the two populations, and after filtering, 1474 and 1381 non-redundant markers of high quality remained for the BC population and the WC population, respectively. A total of 35 and 30 linkage groups were generated for the BC and WC population, respectively, representing all 21 wheat chromosomes. The linkage map of the BC population covered 4094 cM, with an average genetic distance of 2.8 cM between markers, whereas in the WC population 3967 cM covered the genome, with an average marker distance of 2.9 cM. The D genomes had a poorer coverage compared to A and B genomes, especially for chromosomes 3D (10 markers, 87 cM length of the chromosome) and 5D (16, 47 cM) in the BC population and chromosomes 1D (18, 64 cM), 2D (19, 52 cM) and 5D (7, 48 cM) in the WC population, whereas all other chromosomes were represented by larger linkage groups with a length of >100 cM, and mostly >200 cM (Table S1). Of the gene-based markers, *TaCwi-4A* and *Cdex5* were mapped in the BC and WC population, respectively, whereas *Rht-B1* and *Vrn-A1* in both.

### 2.3. QTL Analysis

**Figure 2:**
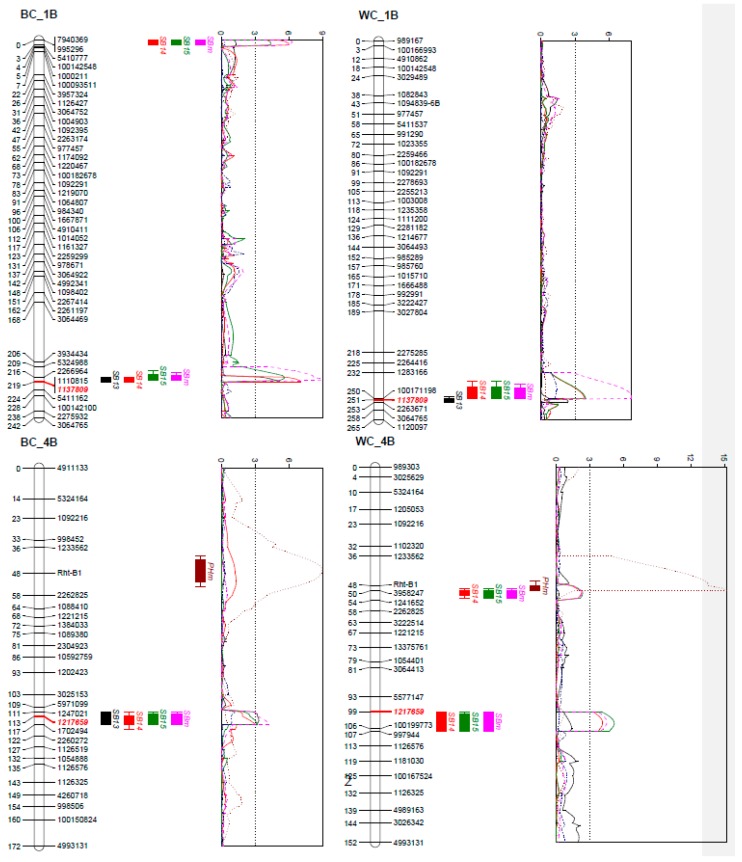

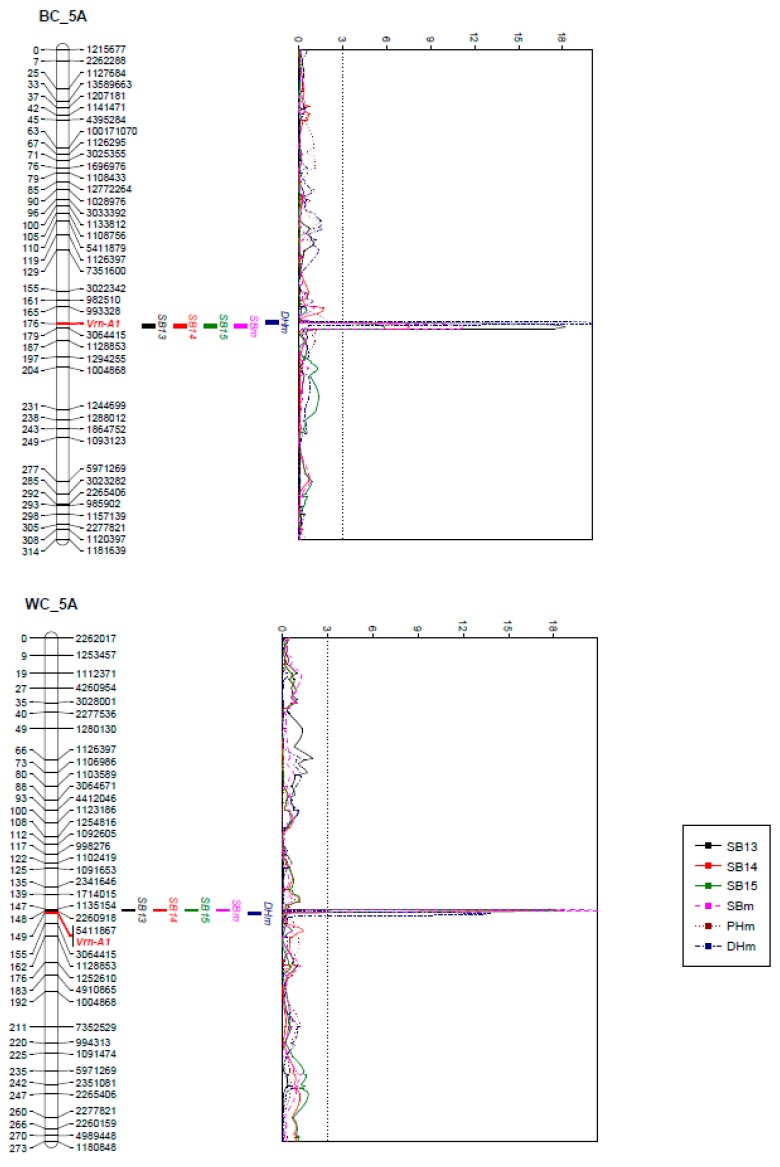
QTL profiles for spot blotch (SB), plant height (PH) and days to heading (DH) on chromosomes 1B, 4B and 5A in the “BARTAI” × “CIANO T79” (BC) and “WUYA” × “CIANO T79” (WC) populations. Genetic distances are shown in centimorgans to the left of the chromosomes. A threshold of 3.0 is indicated by a dashed vertical line in the LOD graphs. Only framework markers are presented except for the QTL regions, and the markers in the QTL regions shared between the two populations are highlighted in red and in Italic.

**Figure 3:**
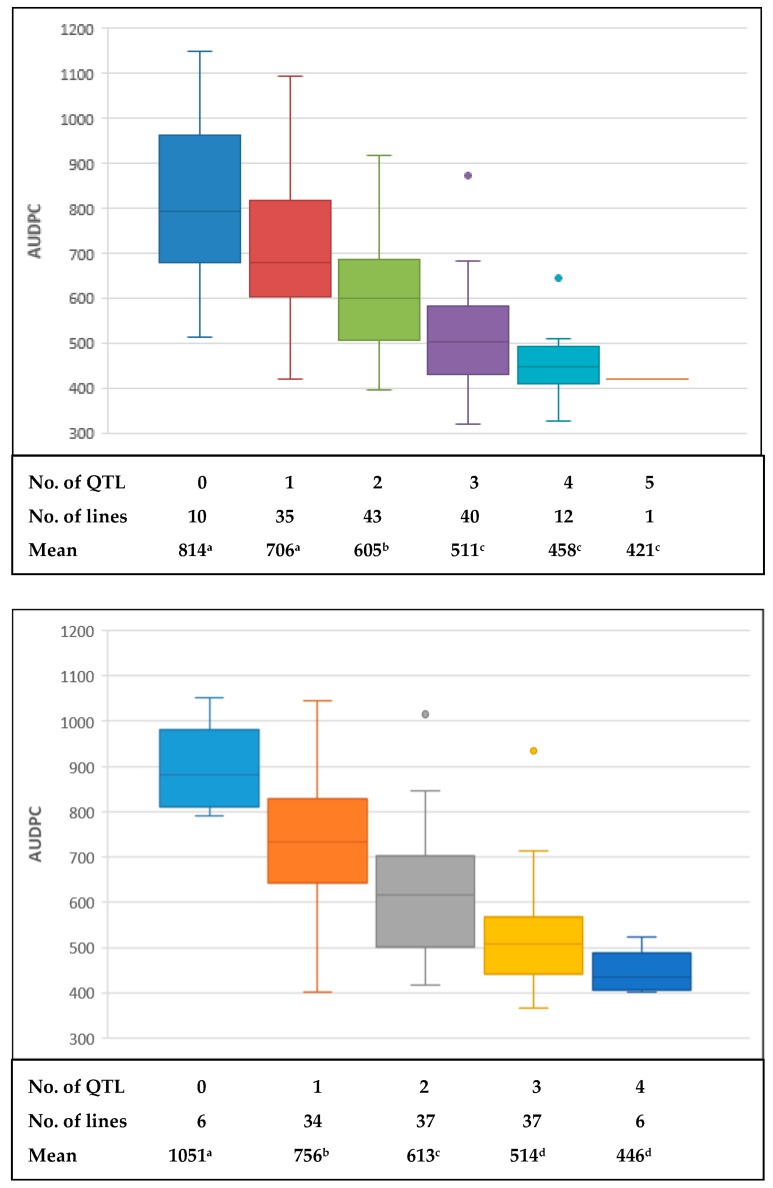
Boxplot for effects of stacking different numbers of QTL in the “BARTAI” × “CIANO T79” (**top**) and “WUYA” × “CIANO T79” (**bottom**) populations. Mean AUDPC values over three experiments were used here. Different letters following mean values of different groups indicate significant difference at alpha = 0.01.

Of the QTL detected in this study, the one on chromosome 5AL located at *Vrn-A1* locus showed the largest phenotypic effects in both populations, explaining phenotypic variations from 6.9 (2015) to 21.9% (2013) in the BC population and from 21.9 (2014) to 27.1% (2013) in the WC population (Table 3 and Figure 2). However, the effects of the QTL on SB reduced substantially when DH and PH were used as covariates (Table S2). A consistent QTL on chromosome 1BL but with lower phenotypic effects was found in both populations (Table 3), and a GBS marker within the QTL regions (1137809) was shared by the two populations, indicating a pair of homologous QTL (Figure 2). The same applied to a QTL on chromosome 4BL (Figure 2). The alleles for resistance were contributed by both parents (Table 3). Additionally, minor QTL from ‘BARTAI’ were found on 4DS (most likely at *Rht-D1*) and 6D in the BC population, from ‘WUYA’ on 2D in the WC population, and from CIANO T79 on 1BS, 1D and 3A in the BC population and on 4B at *Rht-B1* in the WC population (Table 3 and Figure S1). With DH and PH as covariates, additional QTL with minor effects were detected on 3A, 3B, 5B and 7A in the BC population and on 2A in the WC population (Table S2). Phenotypic effects of stacking different numbers of QTL were analysed and the results indicated additive modes of those QTL in both populations (Figure 3). 

## 3. Discussion

Two mapping populations were tested for three years in the field under artificial and natural epiphytotic conditions. We observed nearly continuous distributions of lines in both populations during all three years that indicates the polygenic nature of the disease resistance. The earlier studies also concluded that resistance to spot blotch is rather quantitative [9,10,29]. 

It has long been observed that DH and PH have significant impact on SB resistance, and the general trend is that late and tall lines have more chance to escape the disease [5], which has been repeatedly observed in our previous studies in Mexico [13,23]. In the current study, DH and PH also showed negative correlation with SB severity, although it was not significant in some environments. Further, to confirm the association at a molecular level if any, the QTL mapping results dissected the phenotypic correlation into chromosome regions where SB QTL coincided with those for DH or PH, such as on 4D (*Rht-D1*) and 5A (*Vrn-A1*) in the BC population and 2D (likely *Ppd-D1*), 4B (*Rht-B1*) and 5A (*Vrn-A1*) in the WC population. However, when DH and PH were used as covariates, these QTL either disappeared or became less significant, demonstrating their role as contributor to disease escape. This type of interaction was also found in our research on Fusarium head blight [24,25]. It should be noted that unlike other genes shown above, *Vrn-A1* has major impact on SB resistance in both populations, and its effect often remained significant after DH and PH were used as covariates. It is possible that a true resistance gene is located near *Vrn-A1*, as proposed by Zhu et al. [13], in which a SB resistance QTL was mapped on a chromosome 5AL region outside *Vrn-A1*. If the speculation is true, then the resistance allele of the underlying gene is linked to the *vrn-A1* allele associated with lateness and disease escape, resulting in a genomic region conferring strong SB resistance. In this regard, the functional markers tracing the *vrn*-*A1* allele in marker-assisted selection (MAS) could be very useful to improve SB resistance. If the *Vrn-A1a* allele for reduced vernalization requirement and resulting in earliness is detected in a breeding line, then it will be more likely vulnerable to SB infection, at least in regions like Mexico and South Asia where earliness is mostly associated with higher SB infection [30]. 

In most genetic studies for spot blotch, spry inoculation was used in which inoculum was applied directly onto leaf surface [9,10,11], instead of spreading pathogen-colonized sorghum kernels in the field as described in the current study. The advantage of the former method is to evaluate directly leaf resistance against the pathogen, avoiding the influence of PH and DH. However, under natural SB infection, inoculum comes from soil surface, which spreads from lower leaves upwards until flag leaf [1]. Considering this, the method adopted in the current study mimics better the natural infection process of SB, which is in accordance to disease resistance in field conditions and the QTL identified may thus be more relevant in SB management in large-scale wheat production. In a few other studies, inoculation was conducted at seedling stage to map seedling resistance QTL to SB [17,31]. Although no clear evidence shows the different expression of SB resistance genes at different developmental stages, seedling- and adult plant-specific resistance genes might well exist, explaining why many new QTL have been identified in seedling SB experiments compared to those in adult plant experiments [17,31]. Likewise, the inoculation protocol used in the present study might have also contributed to the identification of a few QTL that have not been reported previously, as discussed below.

A minor but stably expressed QTL on 1BL was identified in both populations. Gurung et al. [18] also reported a QTL on 1BL for SB in an association mapping panel. Comparing the QTL positions of both studies by a BLAST of the marker sequences to the IWGSC RefV1.0 genome sequence of Chinese Spring, indicated the QTL to be the different. The results showed that the QTL in Gurung et al. [18] was in the proximal region of chromosome 1BL, whereas the QTL of this study was in the far distal region of chromosome 1BL. Lillemo et al. [11] reported a SB QTL on 1BL at *Lr46*. The KASP marker for *Lr46* (Lr46_SNP1G22) was, however, monomorphic in both the BC and WC populations, excluding the possibility of the QTL being *Lr46* (also this gene is not on the far distal region of 1BL). Therefore, to the best of our knowledge, the 1BL QTL identified in this study has not been reported earlier and thus is a new QTL for SB resistance. 

Zhu et al. [13] identified a QTL on 1B for SB resistance; based on the SSR markers used in that study, the QTL was on the short arm and its confidence interval overlapped with that of the QTL on 1BS in the BC population, thus the two may be the same QTL. 

The QTL on 4B was located on the long arm. Recently two QTL mapping studies have been published in which QTL on chromosome 4B have been reported. In Ayana et al. [31], the QTL is located on the short arm, while in Jamil et al. [32] the QTL is on the proximal region of what we found in our study, with a physical distance of 89.4 Mb in the IWGSC RefV1.0 genome sequence of Chinese Spring. Thus, the QTL on 4BL found in the current study might be new, though its effect is small and may not be suitable for MAS. Likewise, other small effect QTL listed in Table 3 and Table S1 were often unstable across environments and thus are not suitable for MAS. However, if these QTL could be validated in future studies, associated markers could be used in genomics-assisted breeding. 

Many CIMMYT wheat lines showed highly stable SB resistance, especially the lines included in HLBSNs [23], but their resistance mechanism remained largely unknown. The first insight was derived from the study of Lillemo et al. [11], in which the pleiotropic effect of rust resistance genes *Lr34* and *Lr46* on SB resistance was reported, and the former was named as *Sb1*. Subsequently, Vasistha et al. [33] demonstrated that introgression of *Lr34* can lead to enhanced resistance to SB. The two genes, however, were not mapped in the current study due to the absence of *Lr34* and the fixation of *Lr46* in both populations, making their phenotipic effects cannot be demonstrated here. Nevertheless, the extensive utilization of the two genes in CIMMYT wheat breeding program [34] provides opportunities for improving SB resistance. Another advantage of CIMMYT germplasm on SB resistance is the very low frequency of *Vrn-A1a* [35], which was associated with SB susceptibility as discussed above. Specific to this study, SB resistance was mainly controlled by minor QTL, both detectable and undetectable, and significantly influenced by the *Vrn-A1* region. Reliance on minor gene/QTL based resistance has the advantage of being stable and durable, as demonstrated in rusts, where pyramiding 4–5 minor genes led to “near-immunity” with durable resistance [34]. In spot blotch also, marker-aided backcross breeding was used successfully to enhance resistance in wheat [36]. Based on this theory, stacking *Sb1* (*Lr34*), *Lr46*, *vrn-A1*, which are prevalent in CIMMYT gene pool, and a few more SB resistance QTL, high level of SB resistance is achievable. This would also result in development of durable resistant lines for rusts and spot blotch.

## 4. Materials and Methods 

### 4.1. Plant Materials and Field Trials

Two elite CIMMYT breeding lines, BARTAI (pedigree BABAX/LR42//BABAX/3/ERA F2000) and WUYA (WAXWING * 2/CIRCUS), have shown excellent SB resistance in previous trials. In this study they were used as resistant parents and crossed with the susceptible cultivar CIANO T79 (BUCKY/(SIB)MAYA-74/4/BLUEBIRD//HD-832.5.5/OLESEN/3/CIANO-67/PENJAMO-62) to generate two bi-parental mapping populations. Individual F_2_ were advanced following single seed descend to generate 232 F_2:7_ progenies for each population.

Field experiments were performed at CIMMYT’s Agua Fria station in the State of Puebla, Mexico (altitude 100 m, latitude 20.5° N and longitude 97.6° W). The location has a hot and rainy climate with average annual rainfall of 1200 mm that is very favorable for SB epidemics. Both populations were evaluated in cropping seasons 2012–2013 (denoted as 2013 in this study), 2013–2014 (as 2014) and 2014–2015 (as 2015), sown in November and harvested in March. The plant materials were sown in a randomized complete block design with two replicates. Each plot comprised two rows of one meter with 25-cm spacing, and the plots were separated by a 50-cm space. 

### 4.2. Disease Screening Protocols

A mixture of virulent *B. sorokiniana* isolates was used as field inoculum. The isolates were collected from naturally infected leaf samples at the Agua Fria station and showed similar types and color on culture media. The conserved isolates were reactivated on V8 medium for 5–7 days and then multiplied on sorghum grains previously been soaked and autoclaved. The inoculated sorghum grains were incubated at room temperature for approximately six weeks with frequent shaking to mix the grains and to promote good coverage of the fungus. The infested sorghum grains were then spread in the middle of the double row at Zadoks’ GS29 plant stage [37]. The disease evaluation started at four to five weeks after inoculation, depending on SB development. Visual scoring was done for each plot, using the double-digit scale (00–99) modified from Saari and Prescott’s severity scale for assessing wheat foliar diseases [38]. The first digit (D1) indicates disease progress in canopy height from the ground level and the second digit (D2) stands for severity or proportion of infected leaf area. The SB evaluation was repeated three to four times at weekly intervals. For each evaluation, percentage disease severity was calculated with the following formula:

% severity = (D1/9) × (D2/9) × 100
(1)

The area under disease progress curve (AUDPC) was calculated based on three or four disease evaluations, using the formula:

$$\mathrm{AUDPC}=\sum_{i=1}^{n}[\{(Y_{i}+Y_{\left(i+1\right)})/2\}\times \left(t_{\left(i+1\right)}-t_{i}\right)]$$

where *Y_i_* = SB severity at time *t_i_*, *t*_(*i*+1)_ − *t_i_* = time interval (days) between two disease scores, *n* = number of times when SB was recorded. Mean AUDPC values from two replications in single years, as well as averaged values based on three years’ evaluation, were used for QTL mapping. DH and PH were also scored for the two populations to investigate their association with SB resistance.

### 4.3. Statistical Analyses

The phenotypic data was analysed with the SAS program ver. 9.2. Analysis of variance (ANOVA) was carried out with the PROC GLM module in SAS, and the ANOVA results were used to calculate the broad sense heritability for AUDPC of SB across years, using the formula *h*^2^ = $\sigma_{g}^{2}$/($\sigma_{g}^{2}$ + $\sigma_{g*y}^{2}$/*y* + $\sigma_{e}^{2}$/*ry*), in which $\sigma_{g}^{2}$ stands for genetic variance, $\sigma_{g*y}^{2}$ for genotype-by-year interaction, $\sigma_{e}^{2}$ for error variance, *y* for the number of years, and *r* for the number of replications. Pearson correlation coefficients were calculated with the PROC CORR function in SAS.

### 4.4. Genotyping

Genomic DNA was extracted from young leaves with the CTAB method. The two populations were genotyped with the DArTseq genotyping-by-sequencing (GBS) platform at the Genetic Analysis Service for Agriculture (SAGA) in Guadalajara, Mexico. This genotyping platform is based on a combination of complexity reduction methods developed for array-based DArT and sequencing of resulting representations on next-generation sequencing platforms, as described in details in Li et al. [39]. Additionally, gene-based markers for *Rht-B1*, *Vrn-A1* etc. and a few D-genome specific SNPs using the “Kompetitive Allele Apecific PCR” (KASP) technology (LGC Genomics, Teddington, UK) were deployed [35] (Table S3). Markers with missing data points greater than 20% and segregation ratio beyond the range 0.5–2.0 were excluded from further analysis. Redundant markers were excluded using the BIN functionality of the ICIMapping v. 4.1 software (www.isbreeding.net).

### 4.5. Linkage and QTL Analysis

Linkage groups (LGs) were constructed using JoinMap v.4 software (Kyazma BV, Wageningen, The Netherlands) [40] with LOD values from 3 to 10 and the Maximum Likelihood algorithm for ordering within each LG. Chromosome anchoring of LGs was performed according to the consensus GBS map by Li et al. [39]. The MapQTL v. 6.0 software (Kyazma BV, Wageningen, The Netherlands) [41] was used for QTL mapping, in which interval mapping (IM) was first performed to detect potential QTL for each trait, with subsequent multiple QTL mapping (MQM) for each QTL using the closest linked markers as cofactors. QTL were taken as significant and were reported if they were over the LOD threshold of 3 in at least one environment or over the threshold of 2 in multiple environments. In order to deploy potential QTL that were masked by PH and DH variation, QTL mapping with PH and DH as covariates was also performed. The software MapChart ver. 2.3 [42] was used to draw LGs and LOD curves.

## Figures and Tables

**Table 1:** Analysis of variance and heritability estimates for spot blotch in the “BARTAI” × “CIANO T79” (BC) and “WUYA” × “CIANO T79” (WC) population.

Population	Source	DF	Mean Square	F Value	*p* Value	Heritability
BC	Genotype	231	139905	15.38	<0.0001	0.86
	Year	2	5939021	652.79	<0.0001	
	Genotype × Year	462	19415	2.13	<0.0001	
	Rep (Year)	3	258974	28.47	<0.0001	
	Error	693	9098			
WC	Genotype	230	145412	18.75	<0.0001	0.88
	Year	2	6256059	806.64	<0.0001	
	Genotype × Year	460	17525	2.26	<0.0001	
	Rep (Year)	3	156580	20.19	<0.0001	
	Error	690	7756			

**Table 2:** Pearson correlation coefficients between spot blotch and days to heading or plant height in the “BARTAI” × “CIANO T79” (BC) and “WUYA” × “CIANO T79” (WC) populations across years.

Associated traits	The BC Population			The WC Population		
	2013	2014	2015	2013	2014	2015
Days to heading	−0.49 **	−0.33 **	−0.49 **	−0.47 **	−0.48 **	−0.16
Plant height	−0.12	−0.43 **	−0.13	−0.29 **	−0.49 **	−0.16

** *p* < 0.0001.

**Table 3:** QTL for spot blotch resistance in the “BARTAI” × “CIANO T79” (BC) and “WUYA” × “CIANO T79” (WC) populations and their association with phenological traits.

Population	Linkage Group	Position	Left Marker	Right Marker	2013	2014	2015	Mean	R Source ^a^	Traits Associated ^b^
**BC**	1B	0.4–2.8	995296	5410777		**7.1**	**5.4**	**6.5**	C	
	1B	209.4–218.6	5324988	1110815	**5.5**	**8.5**	**7.0**	**8.9**	B	
	1D	48.4–53.3	100142243	1037975			**3.5**	2.9	C	
	3A	103.2–146.0	1109808	990692	**5.1**			**3.2**	C	
	4B	111.3–117.1	1247021	1702494	**5.2**	2.2	3.0	**3.3**	B	
	4D	0–9.8	BS00036421_51	1119387		**3.6**			B	PH
	5A	175.9–179.4	Vrn-A1	3064415	**21.9**	**8.9**	**6.9**	**12.5**	B	DH
	6D	21.9–33.2	1239681	1095962	2.4	2.6	2.7		B	
	Accumulated percentage of variation explained				40.1	32.9	28.5	37.3		
**WC**	1B	232.3–252.9	1283166	2263671	2.9	**4.4**	**4.5**	**8.3**	W	
	2D	2.0–3.2	1085831	1098973	**7.3**	**4.9**	**4.7**	**9**	W	DH
	4B	47.9–54.1	Rht-B1	1241652		2.6	2.7	2.3	C	PH
	4B	99.2–107.4	1217659	997944		**4.7**	**6**	**4.5**	W	
	5A	147.5–148.4	1135154	2260918	**27.1**	**21.9**	**24.3**	**25.1**	W	DH
	Accumulated percentage of variation explained				37.3	38.5	42.2	49.2		

The percentage of explained phenotypic variation is shown in the table, QTL are listed if they were over the LOD threshold of 3 (in bold) in at least one environment or over the threshold of 2 in multiple environments. **^a^** *B* “BARTAI”, *W* “WUYA”, *C* “CIANO T79”; **^b^** *PH* plant height, *DH* days to heading.

## Supplementary Materials

Supplementary Materials can be found at http://www.mdpi.com/1422-0067/19/12/4054/s1.

## References

[1] Gupta P.K., Chand R., Vasistha N.K., Pandey S.P., Kumar U., Mishra V.K., Joshi A.K. (2018). Spot blotch disease of wheat: The current status of research on genetics and breeding. Plant Pathol..

[2] Duveiller E., Sharma R.C., Sharma I. (2012). Wheat resistance to spot blotch or foliar blight. Disease Resistance in Wheat.

[3] Maraite H., Duveiller E., Dubin H.J., Reeves J., McNab A. (1998). Evolution of the nomenclature used for *Helminthosporium* spp. Causing leaf blight of wheat. Helminthosporium Blights of Wheat: Spot Blotch and Tan Spot.

[4] Raemaekers R.H., Klatt A.R. (1988). Helminthosporium sativum: Disease complex on wheat and sources of resistance in Zambia. Wheat Production Constraints in Tropical Environments.

[5] Duveiller E.M., Sharma R.C. (2009). Genetic improvement and crop management strategies to minimize yield losses in warm non-traditional wheat growing areas due to spot blotch pathogen *Cochliobolus sativus*. J. Phytopathol..

[6] Sharma R., Duveiller E. (2003). Selection index for improving *Helminthosporium* leaf blight resistance, maturity, and kernel weight in spring wheat. Crop. Sci..

[7] Joshi A.K., Ortiz-Ferrara G., Crossa J., Singh G., Alvarado G., Bhatta M.R., Duveiller E., Sharma R.C., Pandit D.B., Siddique A.B. (2007). Associations of environments in South Asia based on spot blotch disease of wheat caused by *Cochliobolus sativus*. Crop. Sci..

[8] Sharma R.C., Duveiller E., Jacquemin J.M. (2007). Microsatellite markers associated with spot blotch resistance in spring wheat. J. Phytopathol..

[9] Kumar U., Joshi A.K., Kumar S., Chand R., Röder M.S. (2009). Mapping of resistance to spot blotch disease caused by *Bipolaris sorokiniana* in spring wheat. Theor. Appl. Genet..

[10] Kumar U., Joshi A.K., Kumar S., Chand R., Röder M.S. (2010). Quantitative trait loci for resistance to spot blotch caused by *Bipolaris sorokiniana* in wheat (*T. aestivum* L.) lines ‘Ning 8201’ and ‘Chirya 3’. Mol. Breed..

[11] Lillemo M., Joshi A.K., Prasad R., Chand R., Singh R.P. (2013). QTL for spot blotch resistance in bread wheat line SAAR co-locate to the biotrophic disease resistance loci *Lr34* and *Lr46*. Theor. Appl. Genet..

[12] Joshi A.K., Chand R., Kumar S., Singh R.P. (2004). Leaf tip necrosis: A phenotypic marker associated with resistance to spot blotch disease in wheat. Crop. Sci..

[13] Zhu Z., Bonnett D., Ellis M., Singh P., Heslot N., Dreisigacker S., Gao C., Mujeeb-Kazi A. (2014). Mapping resistance to spot blotch in a CIMMYT synthetic-derived bread wheat. Mol. Breed..

[14] Kumar S., Röder M.S., Tripathi S.B., Kumar S., Chand R., Joshi A.K., Kumar U. (2015). Mendelization and fine mapping of a bread wheat spot blotch disease resistance QTL. Mol. Breed..

[15] Lu P., Liang Y., Li D., Wang Z., Li W., Wang G., Wang Y., Zhou S., Wu Q., Xie J. (2016). Fine genetic mapping of spot blotch resistance gene *Sb3* in wheat (*Triticum aestivum*). Theor. Appl. Genet..

[16] Singh V., Singh G., Chaudhury A., Ojha A., Tyagi B.S., Chowdhary A.K., Sheoran S. (2016). Phenotyping at hot spots and tagging of QTLs conferring spot blotch resistance in bread wheat. Mol. Biol. Rep..

[17] Adhikari T.B., Gurung S., Hansen J.M., Jackson E.W., Bonman J.M. (2012). Association mapping of quantitative trait loci in spring wheat landraces conferring resistance to bacterial leaf streak and spot blotch. Plant Genome.

[18] Gurung S., Mamidi S., Bonman J.M., Xiong M., Brown-Guedira G., Adhikari T.B. (2014). Genome-wide association study reveals novel quantitative trait loci associated with resistance to multiple leaf spot diseases of spring wheat. PLoS ONE.

[19] McDonald M.C., Ahren D., Simpfendorfer S., Milgate A., Solomon P.S. (2017). The discovery of the virulence gene *ToxA* in the wheat and barley pathogen *Bipolaris sorokiniana*. Mol. Plant Pathol..

[20] Friesen T.L., Holmes D.J., Bowden R.L., Faris J.D. (2018). *ToxA* is present in the U.S. *Bipolaris sorokiniana* population and is a significant virulence factor on wheat harboring *Tsn1*. Plant Dis..

[21] Joshi A.K., Chand R. (2002). Variation and inheritance of leaf angle, and its association with spot blotch (*Bipolaris sorokiniana*) severity in wheat (*Triticum aestivum*). Euphytica.

[22] Joshi A.K., Chand R., Arun B. (2002). Relationship of plant height and days to maturity with resistance to spot blotch in wheat. Euphytica.

[23] Singh P.K., Zhang Y., He X., Singh R.P., Chand R., Mishra V.K., Malaker P.K., Reza M.A., Rahman M.M., Islam R. (2015). Development and characterization of the 4th CSISA-spot blotch nursery of bread wheat. Eur. J. Plant Pathol..

[24] He X., Lillemo M., Shi J., Wu J., Bjørnstad Å., Belova T., Dreisigacker S., Duveiller E., Singh P. (2016). QTL characterization of Fusarium head blight resistance in CIMMYT bread wheat line Soru#1. PLoS ONE.

[25] He X., Singh P.K., Dreisigacker S., Singh S., Lillemo M., Duveiller E. (2016). Dwarfing genes *Rht-B1b* and *Rht-D1b* are associated with both type I FHB susceptibility and low anther extrusion in two bread wheat populations. PLoS ONE.

[26] Brown J.K.M., Chartrain L., Lasserre-Zuber P., Saintenac C. (2015). Genetics of resistance to *Zymoseptoria tritici* and applications to wheat breeding. Fungal Genet. Biol..

[27] Dubin H., Rajaram S. (1996). Breeding disease-resistant wheats for tropical highlands and lowlands. Annu. Rev. Phytopathol..

[28] Mujeeb-Kazi A., Villareal R., Gilchrist L., Rajaram S. (1996). Registration of five wheat germplasm lines resistant to *Helminthosporium* leaf blight. Crop. Sci..

[29] Joshi A.K., Kumar S., Chand R., Ortiz-Ferrara G. (2004). Inheritance of resistance to spot blotch caused by *Bipolaris sorokiniana* in spring wheat. Plant Breed..

[30] Saxesena R.R., Mishra V.K., Chand R., Chowdhury A.K., Bhattacharya P.M., Joshi A.K. (2017). Pooling together spot blotch resistance, high yield with earliness in wheat for eastern Gangetic plains of south Asia. Field Crop. Res..

[31] Ayana G.T., Ali S., Sidhu J.S., Gonzalez Hernandez J.L., Turnipseed B., Sehgal S.K. (2018). Genome-wide association study for spot blotch resistance in hard winter wheat. Front. Plant Sci..

[32] Jamil M., Ali A., Gul A., Ghafoor A., Ibrahim A.M.H., Mujeeb-Kazi A. (2018). Genome-wide association studies for spot blotch (*Cochliobolus sativus*) resistance in bread wheat using genotyping-by-sequencing. Phytopathology.

[33] Vasistha N.K., Balasubramaniam A., Mishra V.K., Srinivasa J., Chand R., Joshi A.K. (2017). Molecular introgression of leaf rust resistance gene *Lr34* validates enhanced effect on resistance to spot blotch in spring wheat. Euphytica.

[34] Singh R.P., Huerta-Espino J., Bhavani S., Herrera-Foessel S.A., Singh D., Singh P.K., Velu G., Mason R.E., Jin Y., Njau P. (2011). Race non-specific resistance to rust diseases in CIMMYT spring wheats. Euphytica.

[35] Dreisigacker S., Sukumaran S., Guzmán C., He X., Lan C., Bonnett D., Crossa J., Rajpal V., Rao S., Raina S. (2016). Molecular marker-based selection tools in spring bread wheat improvement: CIMMYT experience and prospects. Molecular Breeding for Sustainable Crop Improvement.

[36] Vasistha N.K., Balasubramaniam A., Mishra V.K., Chand R., Srinivasa J., Yadav P.S., Joshi A.K. (2015). Enhancing spot blotch resistance in wheat by marker-aided backcross breeding. Euphytica.

[37] Zadoks J.C., Chang T.T., Kozak C.F. (1974). A decimal code for the growth stages of cereals. Weed Res..

[38] Saari E.E., Prescott J.M. (1975). A scale for appraising the foliar intensity of wheat disease. Plant Dis. Rep..

[39] Li H., Vikram P., Singh R.P., Kilian A., Carling J., Song J., Burgueno-Ferreira J.A., Bhavani S., Huerta-Espino J., Payne T. (2015). A high density gbs map of bread wheat and its application for dissecting complex disease resistance traits. BMC Genom..

[40] Van Ooijen J. (2006). JoinMap® 4, Software for the Calculation of Genetic Linkage Maps in Experimental Populations.

[41] Van Ooijen J. (2009). MapQTL® 6, Software for the Mapping of Quantitative Trait Loci in Experimental Populations of Diploid Species.

[42] Voorrips R.E. (2002). Mapchart: Software for the graphical presentation of linkage maps and QTLs. J. Hered..

