# Supplementary material for: Resistance to Spot Blotch in Two Mapping Populations of Common Wheat Is Controlled by Multiple QTL of Minor Effects
Source: Int J Mol Sci. 2018 Dec 14;19(12):4054. doi: 10.3390/ijms19124054 (PMC6321084; doi:10.3390/ijms19124054)
Supplement: Supplementary file 1 [file ijms-19-04054-s001.pdf]

Table S1. QTL for spot blotch resistance in the 'BARTAI' × 'CIANO T79' (BC) and 'WUYA' × 'CIANO T79' (WC) populations, using days to heading and plant height as covariates

	Linkage group	Position	Left marker	Right marker	2013	2014	2015	Mean	R source[a]
**BC**	1B	0.4-5.5	995296	1000211	1.8	**7.4**	**6.7**	**8.3**	C
	1B	215.8-218.6	2266964	1110815	**3.5**	**6.2**	**5.9**	**4.7**	B
	3A	103.2-146.0	1109808	990692	**4.9**				C
	3AL	28.8-32.0	987922	100199979	2.3	2		**2.3**	B
	3B	65.6-71.3	1089285	1091357			**4.1**	**2.5**	B
	5A	175.9-179.4	Vrn-A1	3064415	**9.7**	**3.9**		**2.2**	B
	5B	142.1-142.9	3028824	1108969			**3.2**		B
	6D	4.3-33.2	5324990	1095962		1.8	**3.4**	2	B
	7A2	8.3-12.8	1008884	1079791	**3.1**			1.5	C
	Accumulated percentage of variation explained				12.5	15.6	16.7	20	
**WC**	1B	232.3-252.9	1283166	2263671	2.2	**8.3**	2.5	**5.1**	W
	2A	110.5-113.5	5579629	1094923	3			1.9	W
	2D	1.1-3.2	9724392	1098973	2.8	**3.1**		**2.4**	W
	4B	95.7-106.3	1092405	100199773		**2.4**		**3.5**	W
	5A	145.7-158.7	100168681	1049519	**12.5**	**6.6**	**6.8**	**6.3**	W
	Accumulated percentage of variation explained				20.5	20.4	9.3	19.2	

The percentage of explained phenotypic variation is shown in the table, QTL are listed if they were over the LOD threshold of 3 (in bold) in at least one environment or over the threshold of 2 in multiple environments. [a] *B* 'BARTAI', *W* 'WUYA', *C* 'CIANO T79'

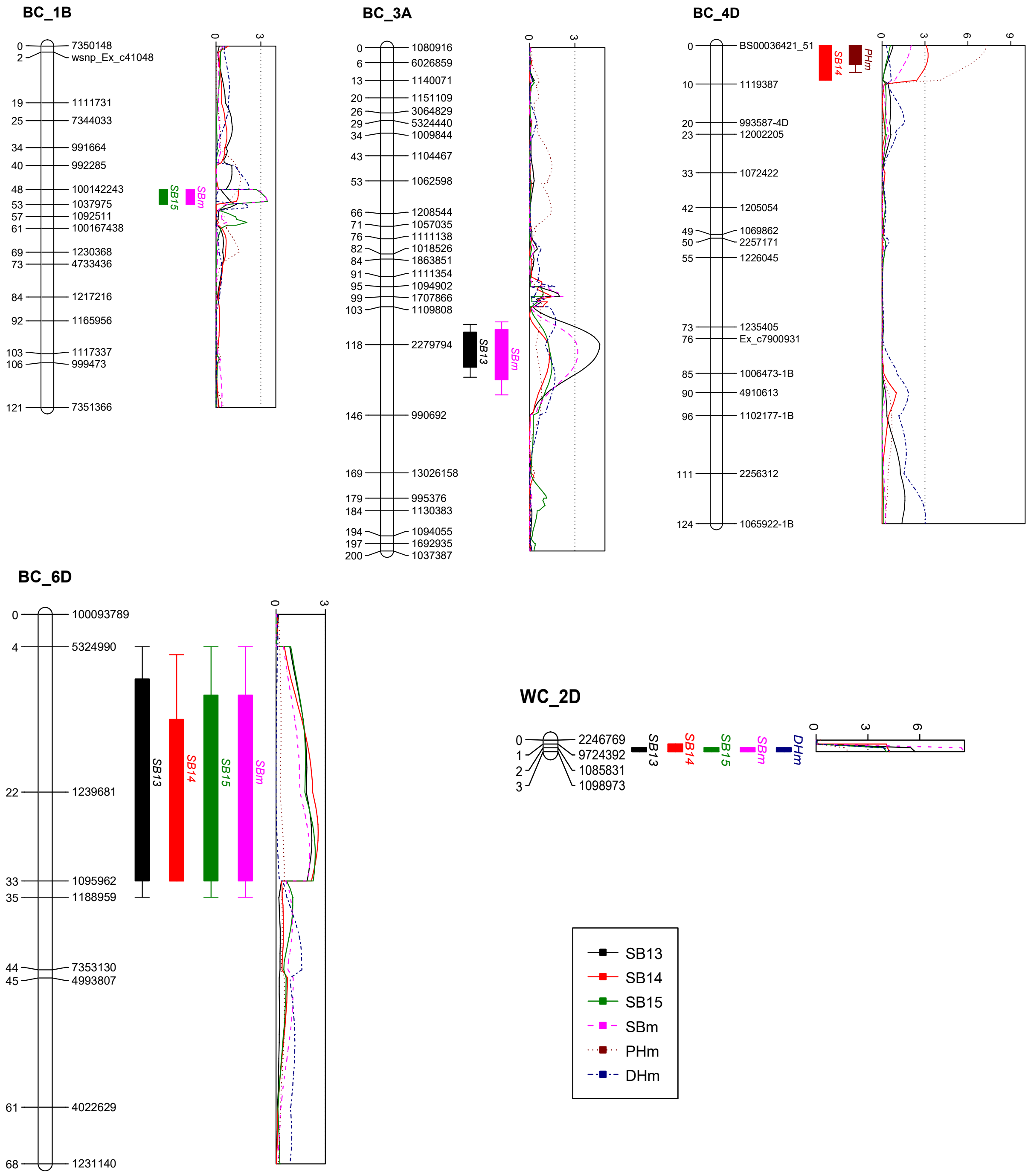

Figure S1. QTL profiles for spot blotch (SB), plant height (PH) and days to heading (DH) on chromosomes 1B, 3A, 4D and 6D in the 'BARTAI' × 'CIANO79' (BC) population and on chromosome 2D in the 'WUYA' × 'CIANO79' (WC) population. Genetic distances are shown in centimorgans to the left of the chromosomes. A threshold of 3.0 is indicated by a dashed vertical line in the LOD graphs. Only framework markers are presented except for the QTL regions.
